# The *Echinococcus granulosus* Antigen B Gene Family Comprises at Least 10 Unique Genes in Five Subclasses Which Are Differentially Expressed

**DOI:** 10.1371/journal.pntd.0000784

**Published:** 2010-08-10

**Authors:** Wenbao Zhang, Jun Li, Malcolm K. Jones, Zhuangzhi Zhang, Li Zhao, David Blair, Donald Peter McManus

**Affiliations:** 1 Molecular Parasitology Laboratory, Infectious Diseases Division, Queensland Institute of Medical Research, Brisbane, Queensland, Australia; 2 Xinjiang Veterinary Research Institute, Xinjiang Academy of Animal Science, Urumqi, Xinjiang, China; 3 School of Veterinary Science, University of Queensland, Brisbane, Queensland, Australia; 4 School of Marine and Tropical Biology, James Cook University, Townsville, Queensland, Australia; Rosetta Genomics, Israel

## Abstract

**Background:**

Antigen B (EgAgB) is a major protein produced by the metacestode cyst of *Echinococcus granulosus,* the causative agent of cystic hydatid disease. This protein has been shown to play an important role in modulating host immune responses, although its precise biological function still remains unknown. It is generally accepted that *EgAgB* is comprised of a gene family of five subfamilies which are highly polymorphic, but the actual number of genes present is unknown.

**Methodology/Principal Findings:**

Based on published sequences for the family, we designed specific primers for each subfamily and used PCR to amplify them from genomic DNA isolated from individual mature adult worms (MAW) taken from an experimentally infected dog in China and individual larval protoscoleces (PSC) excised from a single hydatid cyst taken from an Australian kangaroo. We then used real-time PCR to measure expression of each of the genes comprising the five *EgAgB* subfamilies in all life-cycle stages including the oncosphere (ONC).

**Conclusions/Significance:**

Based on sequence alignment analysis, we found that the *EgAgB* gene family comprises at least ten unique genes. Each of the genes was identical in both larval and adult *E. granulosus* isolates collected from two geographical areas (different continents). DNA alignment comparisons with *EgAgB* sequences deposited in GenBank databases showed that each gene in the gene family is highly conserved within *E. granulosus*, which contradicts previous studies claiming significant variation and polymorphism in *EgAgB*. Quantitative PCR analysis revealed that the genes were differentially expressed in different life-cycle stages of *E. granulosus* with *EgAgB3* expressed predominantly in all stages. These findings are fundamental for determining the expression and the biological function of antigen B.

## Author Summary

Antigen B (EgAgB) is a major protein produced by the metacestode cyst of *Echinococcus granulosus* and plays an important role in modulating host immune responses, although its precise biological function still remains unknown. Previous studies suggested the *EgAgB* gene family is variable between isolates and genotypic strains of *E. granulosus*. We designed specific primers to amplify and determine the number and variation of the genes using genomic DNA from individual worms. Based on sequence alignment analysis, we found that the gene family comprises ten unique genes. Each of the genes was identical in both larval and adult *E. granulosus* and in isolates collected from the two distinct geographical areas. We showed that the genes were differentially expressed in different stages of *E. granulosus* with one gene, *EgAgB3/1,* expressed predominantly in all stages. This is the first study to report such a large number of unique and conserved genes in the *EgAgB* gene family and their differential expression in different life cycle stages of *E. granulosus*. These findings are fundamental for determining the expression and regulation of this gene family in *E. granulosus* and the biological function of antigen B.

## Introduction

Antigen B (EgAgB) is the most abundant protein generated by the pathogenic larval stage (hydatid cyst or metacestode) of *Echinococcus granulosus,* the cause of cystic echinococcosis (CE). Synthesized and secreted by both cyst germinal layer and protoscoleces [1], the protein is highly immunogenic and can be recognised by more than 80% of sera from patients with CE [2], [3]. Nevertheless, its precise biological function remains undetermined, although one report suggests that EgAgB might have lipid-binding properties [4]. It has been as well hypothesised that EgAgB plays a key role in the interaction between parasite and host based on studies showing it functions as a serine protease inhibitor that impairs neutrophil chemotaxis [5] and as an immune modulator that skews Th1/Th2 cytokine ratios to Th2 polarized responses [6], benefiting parasite survival in the mammalian host [7]. A number of previous studies have also indicated that the protein is encoded by a gene family [8], that is highly variable between isolates and strains of *E. granulosus* [5], [8]–[10].

We believe the high levels of variation reported by others was based on comparisons of paralogs, amplified using conserved primers and assumed to be orthologs. Until now, there have been no data showing how many genes are represented in the *EgAgB* family, although it is known that there are five subfamilies (*EgAgB1-5*) present [5], [8], [9], [11]–[13]. Genomic Southern blots revealed that the gene family should include at least seven genes [14]. However, as these genes are highly similar, especially at the subfamily level, it has proven difficult to generate clear data from the Southern blot analysis. Determining the number of the genes in the family is fundamental for further exploring the expression and regulation of *EgAgB* in *E. granulosus.* This will provide insight to more fully understanding its biological function in this and other taeniid species, which share similar gene sequences to those found in *E. granulosus* [15]–[18].

We cloned and sequenced ten unique genes from individual worms (adults and protoscoleces) of *E. granulosus* and show that each is conserved in parasites originating from different geographical areas and hosts. Further, we show the differential expression of all of the family of genes in five developmental stages of *E. granulosus* by real time PCR and cDNA sequencing.

## Materials and Methods

### Extraction of genomic DNA from individual protoscoleces and adult worms of *E. granulosus*

Protoscoleces (PSC) of *E. granulosus* were aspirated from a fertile hydatid cyst collected from a kangaroo (*Macropus giganteus*) from eastern Australia. The cyst was kindly provided by Dr. Tamsin Barnes from a previous study [19]. Mature adult worms (MAW) were collected from a dog from Xinjiang, China [20]. The parasite materials were stored until use in liquid nitrogen as described [20].

PSC and MAW were thawed in RNAlater (Ambion, Austin, USA) and diluted with water. Individual PSC and MAW were respectively pipetted into plastic mortar microtubes (Sigma–Aldrich, St. Louis, USA) under microscopy to make sure that each tube contained a single parasite. After a brief centrifugation to spin-down the parasite, 50 µl of PrepMan Ultra Sample Preparation Reagent (Applied Biosystems, Foster, USA) was added to each of the tubes. The single parasite was ground with a micro-grinder using a plastic pestle. The homogenate was heated at 100°C for 10 min and centrifuged at 16,000 g for 5 min. The supernatant was precipitated with 1× vol of isopropanol. The invisible pellet was washed with 70% (v/v) ethanol, dissolved in 50 µl water and used as DNA template.

### PCR to amplify *E. granulosus* antigen B genes

PCR reactions were performed with a Taq polymerase kit (Promega, Madison, WI) with 5 µl of the DNA template preparation and 20 pmol of each PCR primer in a final volume of 50 µl. To amplify the *EgAgB* gene fragments from genomic DNA, we designed two forward primers, EgAgBF1, specific for subfamily *EgAgB*1 and *EgAgB*3, and EgAgBF2, specific for subfamily *EgAgB*2, *EgAgB*4 and *EgAgB*5 based on previous studies [8], [9], [11],[12],[21]. The forward primers were based on the first exonic sequences of the *EgAgB* gene family. We designed eight down-stream primers, which were specific for each of the gene subfamilies (the primers for *EgAgB1* and *EgAgB3* were within the second exons). All primers used to isolate the *EgAgB* gene variants are listed in Table 1. Amplification was performed with 35 cycles of 94°C for 30 s, 54°C for 30 s and 72°C for 30 s, followed by a denaturing step at 94°C for 1 min, and a final extension step at 72°C for 7 min on a Mastercycle Gradient thermocycler (Eppendorf, Hamburg, Germany).

**Table 1:** Primers used to amplify genes of the *EgAgB* gene family.

Name	Sequence 5′-3′	Specific for:	Product size (bp)
EgAgBF1	TCTCGCTCTGGCTCTCGTC	*EgAgB1* and *3*	-
EgAgBF2	TCTCTCTCTTGCTCTCGTGGC	*EgAgB2, 4* and *5*	-
EgAgB1/1R	CTTCAGCAATCAACCCTCTGA	*EgAgB1/1*	315*
EgAgB2/1R	TGAATCATCATCTTTTTCTTCCACC	*EgAgB2*	318†
EgAgB3R	TGCCTTCTTCCTCACCATCTC	*EgAgB3/1*–*4*	351, 336,342, 339*
EgAgB4/1R	GAATCATCCTCTTCTTCTTCCTCTTC	*EgAgB4/1*	353†
EgAgB4/2R	CTACTTTGAATCATCATCTTCTTCTTCC	*EgAgB4/2*	360†
EgAgB4/3R	CTTTAAATCATCCTCATCTTCTTCTTCC	*EgAgB4/3*	358†
EgAgB5/1R	CCTTCATCCATCAACCTTTTGAC	*EgAgB5*	334†
EgAgB24UTR	CAAATCATGTGTCCCGACGCATG	*EgAgB2* and *4*	342–387†

*with EgAgBF1 as forward primer.

†with EgAgBF2 as forward primer.

### Cloning and sequencing

PCR products were purified using PCR Purification Kits (Qiagen, Hilden, Germany). Fifty ng of the PCR products were ligated with 50 ng of pGEM-T vector (Promega) in a final volume of 20 µl according to the manufacturer's instructions. One microlitre of the ligation reaction was used to transform 20 µl of competent *E. coli* strain JM109 cells (Promega). White colonies containing inserts were selected on LB agar plates containing ampicillin and X-gal. As each pair of primers is specific to each subfamily, and may amplify gene fragments with different sized PCR products, a quick plasmid extraction/PCR step was performed to determine the size of inserts before selecting clones for sequencing. In brief, after the white colonies had grown to about 0.5 mm in diameter, 30–50 of these colonies from each transformation were individually transferred to microtubes containing 50 µl of water. After vortexing and centrifugation at 12,000 g for 1 min, 10 µl of the supernatant from each tube was used as DNA template for PCR using the same original primers. For each transformation, 3–10 colonies with the same sized insert were selected for sequencing, performed using a Big-Dye Version 3.0 kit on an ABI 377 sequencer (Applied Biosystems) after purification with QIAprep Spin Miniprep Kits (Qiagen).

### Sequence analysis

Inspection of the amino-acid sequences inferred from data collected during this study and obtained from the public databases showed that some members of the *EgAgB* subfamily could be aligned with ease. However, sequences from other subfamilies of *EgAgB* and sequences from other cestodes proved more difficult to align. Furthermore any alignment would be short: 54 amino acids being the length of the shortest sequence. However, to produce a graphical representation of the data, we constructed a simple phylogenetic tree to show the different clusters clearly, including relationships among members of each subfamily. We accept that such a tree does not provide robust inference for the deeper nodes. Bioedit (http://www.mbio.ncsu.edu/BioEdit/bioedit.html) was used to align sequences. Molecular Evolutionary Genetics Analysis version 4 (MEGA v4) [22] program (http://www.megasoftware.net/) was used to construct the tree from amino acid sequences translated from the second exonic sequences of *EgAgB* amplified and cloned from *E. granulosus* in this study and homologous protein sequences from other cestode parasites deposited in the GenBank, EMBL and DDBJ databases, after removal of the signal peptides at their N terminal. A distance matrix was constructed using a Poisson correction method before a mid-point rooted tree was constructed by the minimum-evolution method. One thousand bootstrap cycles were used.

### Extraction of total RNA and RT- PCR

We used quantitative PCR to determine the expression level of each of the *EgAgB* family of genes in five life cycle stages/structural compartments of the cyst of *E. granulosus*. These were: protoscolex (PSC), cyst germinal membrane (CM), immature adult worm (IAW), mature adult worm (MAW) and oncosphere (ONC). Sheep livers containing hydatid cysts were collected from a slaughterhouse in Urumqi, Xinjiang, China. The inner parasite cyst membrane was carefully released from the outer host capsule under sterile conditions. PSC and brood capsules containing PSC were aspirated and then treated with 1% (w/v) pepsin in saline, pH 3 [23], to remove capsule membranes and immature PSC. After 3 washes, the precipitated PSC were stored in liquid nitrogen until use. To prepare the CM, the inner cyst membrane was rinsed several times with PBS to remove any remaining PSC, and the membrane was divided into small pieces. These were pooled and stirred at 4°C for 30 min to release the germinal layer from the laminated layer. After leaving 1 min on ice, the laminated membranes were precipitated and the supernatant transferred to a fresh tube. After centrifugation at 3000 g at 4°C for 15 min, the pellet (CM) contained mostly germinal cells that were stored in liquid nitrogen until use. IAW and MAW (from dogs infected with sheep PSC) and activated ONC were prepared as described [20], [24].

Total RNA was extracted from the different stages/compartments of *E*. *granulosus* using TRIzol reagent (Invitrogen, Carlsbad, CA, USA), according to the supplier's instructions. The RNA was treated with DNase I (Promega) to remove possible genomic DNA contamination. All the RNA samples were of high quality (A260/A280 nm>1.8 and <2.0 in nuclease-free water) assessed using a Bioanalyzer RNA Pico LabChip (Bioanalyer). First-strand cDNA synthesis was carried out with oligo (dT) 12–18 using a Superscript Reverse Transcription kit (Qiagen) with 45 ng of total RNA, according to the manufacturer's instructions. For real time PCR, all cDNA samples were diluted to a concentration of10 ng/µl. Subsequently, 5 µl aliquots were combined with 10 µl of SYBR Green, 3 µl of water and 2 µl (5 pmol) of the forward and reverse primers listed in Table S1. Each experiment was performed in triplicate. Expression profiles of *EgAgB1-5* in the different stages/compartments were obtained by real time PCR using a Rotor Gene (6000) real time PCR machine (Qiagen) and data were analysed by Rotor Gene 6 Software. To identify the expression profile of EgAgB3, we used a pair of primers, EgAgBF1 and EgAgB3R (Table 1), to amplify cDNAs obtained by reverse transcription from total RNA isolated from the five *E. granulosus* stages/compartments. The resulting PCR products were ligated into pGEM-T (Promega) and then transformed into *E. coli* strain JM109. We randomly selected 30 colonies from each of the transformations for sequencing.

## Results

### A single *E. granulosus* worm contains ten genes of the *EgAgB* family

**Figure 1:**
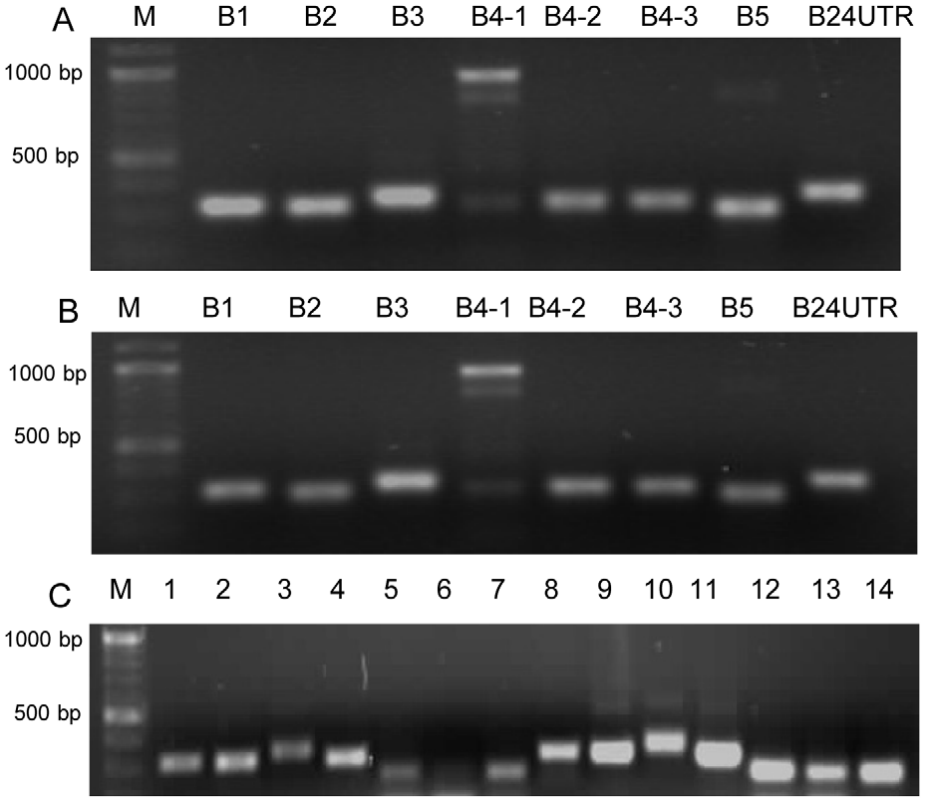
PCR to amplify *E. granulosus* antigen B genes from individual worms. DNA fragments were amplified by PCR with different primer combinations (Table 1) using genomic DNA as templates which were isolated from a single MAW (panel **A**) and a single PSC (panel **B**). Lane B1, *EgAgB1*; lane B2, *EgAgB2*; Lane B3, *EgAgB3*; Lane B4-1, *EgAgB4/1*; Lane B4-2, *EgAgB4/2*; Lane B4-3, *EgAgB4/3;* Lane B5, *EgAgB5*; Lane B24, PCR products amplified with EgAgBF2 and EgAgB24URT, which was designed based on 3′ untranslated region sequences of *EgAgB2* and *EgAgB4*. Panel **C**, Examples showing rapid PCR to determine the size of inserts before sequencing. Lane 1–4, EgAgB1–4 clones from PSC (ZGP5); lane 5–7, EgAgB3 clones from ZGP5 containing the second exon fragments; lane 8–11, EgAgB1–4 clones from MAW (ZGA2); lane 12–14, EgAgB3 clones from ZGA2 containing the second exon fragments. DNA makers (M) are shown to the left of the panels.

**Figure 2:**
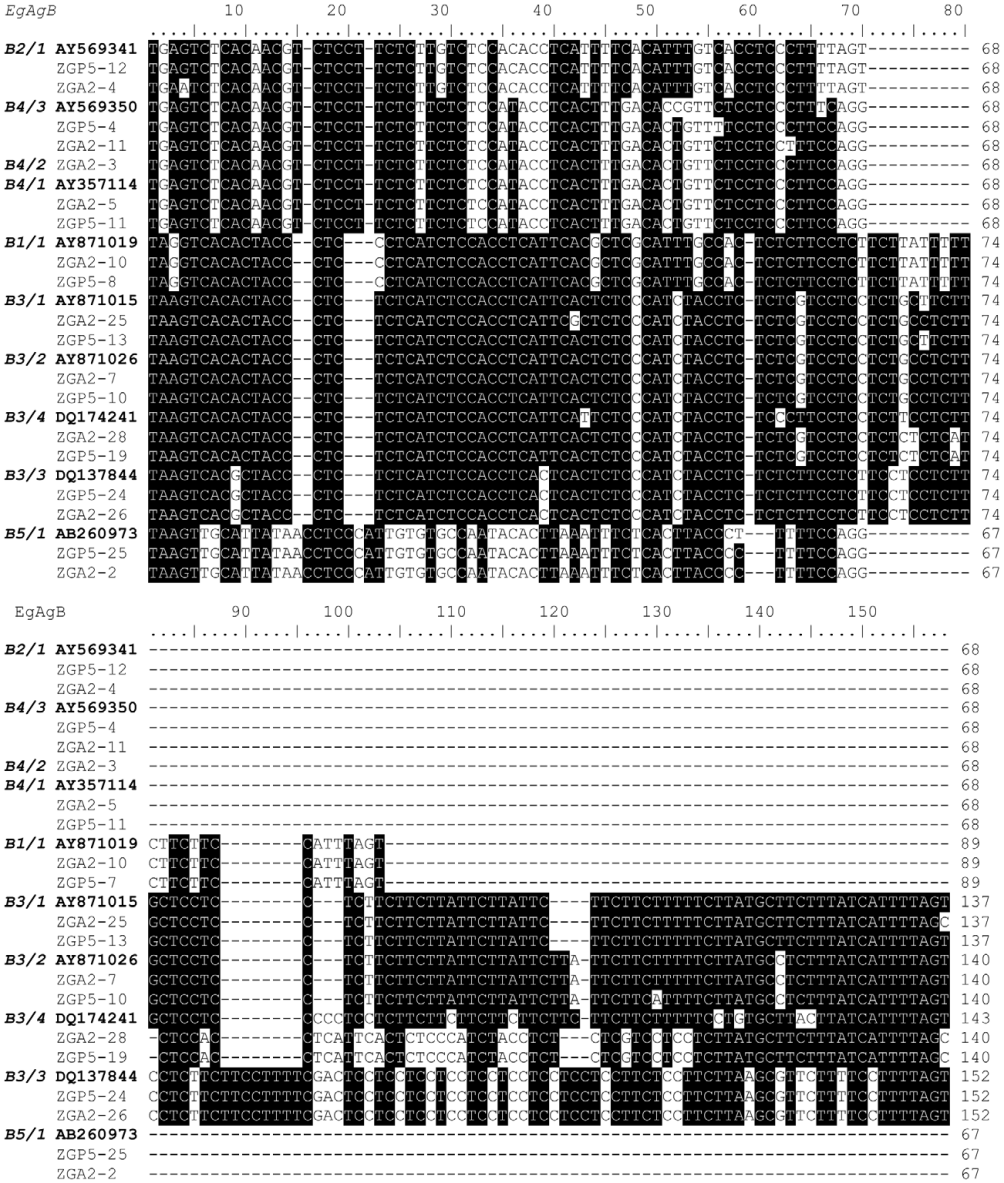
Alignment comparison of intronic sequences of the *E. granulosus* antigen B family. DNA sequences were isolated from a single PSC (ZGP5) and a single MAW (ZGA2) compared with representative antigen B sequences deposited in the GenBank databases. Subfamilies 1, 2 and 5 each consist of one gene, termed *EgAgB1/1* (accession no. **AY871019**), *EgAgB2/1* (**AY569341**) and *EgAgB5/1* (**AB260973**), indicated by B1/1, B1/2 and B5/1, respectively; subfamily 3 has four genes, named *EgAgB3/1* (**AY871026**), *EgAgB3/2* (**AY871015**), *EgAgB3/3* (**DQ137844**) and *EgAgB3/4* (**DQ174241**), indicated by B3/1, B3/2, B3/3 and B3/4, respectively; and subfamily 4 has three genes, named *EgAgB4/1* (**AY357114**), *EgAgB4/2* (**AY569350**) and *EgAgB4/3* (**AF252859**) indicated by B4/1, B4/2 and B4/3, respectively. Homologies are assigned with black representing identity in at least six sequences and white representing different nucleotides. Missing or unmatched sequence is hyphenated.

**Figure 3 pntd-0000784-g003:**
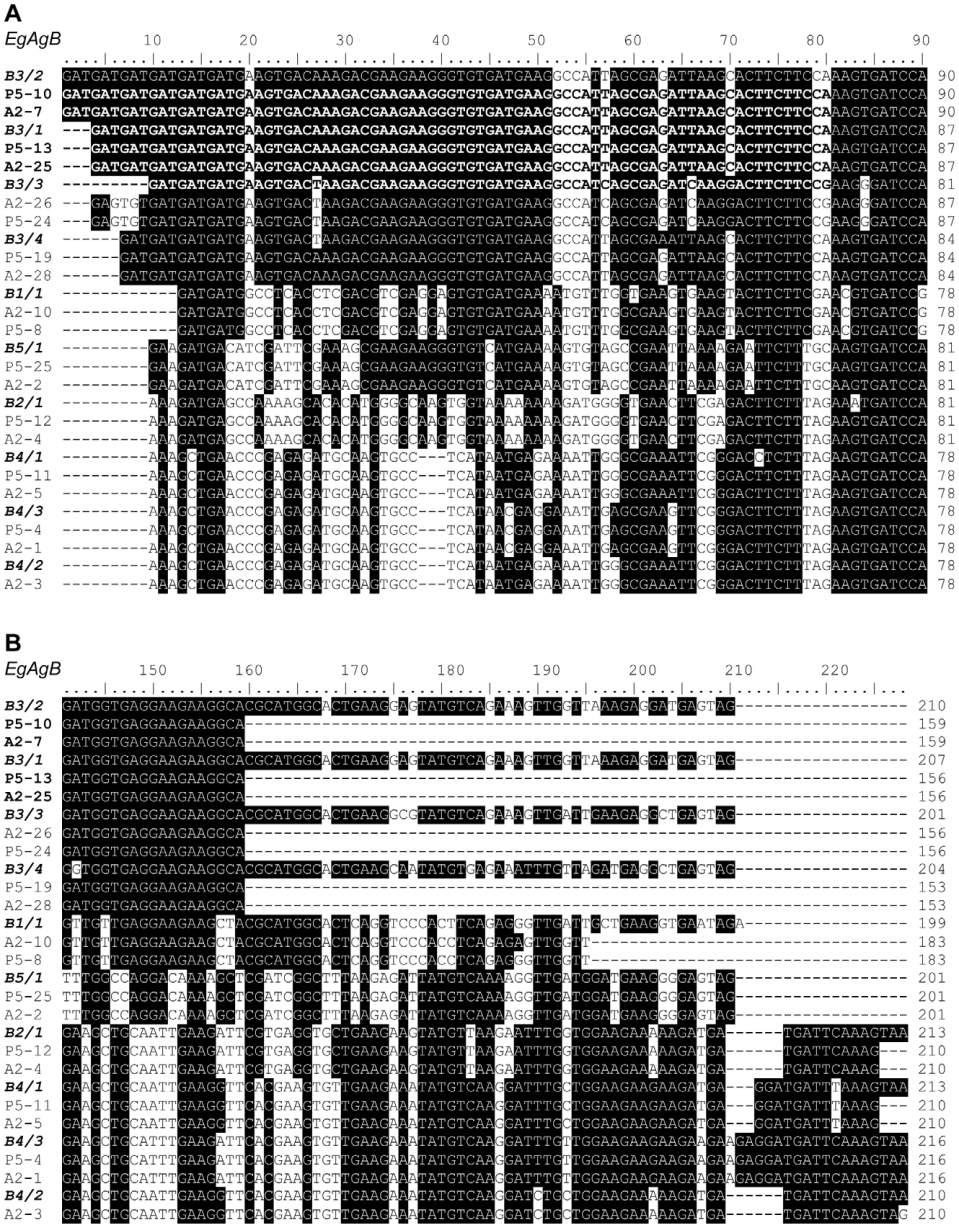
Alignment comparison of the second exons of the *E. granulosus* antigen B family. DNA sequences were isolated from a single PSC (ZGP5) and a single MAW (ZGA2) compared with representative antigen B sequences deposited in the GenBank databases. Homologies are assigned with black representing identity in at least sic sequences and white representing different nucleotides. Missing or unmatched sequence is hyphenated.

**Figure 4:**
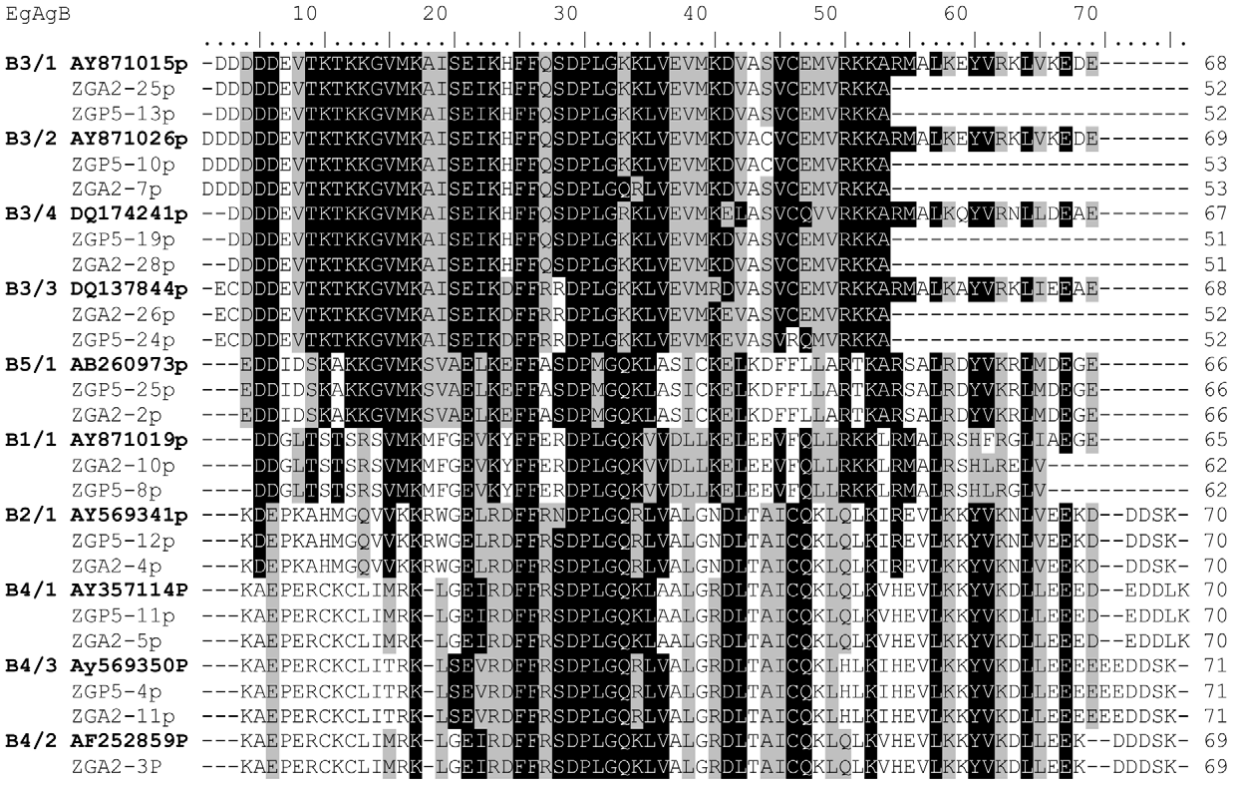
Alignment comparison of amino acid sequences of the *E. granulosus* antigen B family. Proteins sequences were predicted from the second exons of *E. granulosus* antigen B shown in Figure 3 and compared with the representative sequences deposited in the GenBank databases. Homologies are assigned with black representing identity in at least six sequences with white representing different nucleotides. Missing or unmatched sequence is hyphenated.

With the eight combinations of primers shown in Table 1, we successfully amplified gene fragments with genomic DNA extracted from six individual MAW isolated from a dog (from China) and five individual PSC isolated from a single cyst from a kangaroo (from Australia). Fig. 1 shows representative examples of the amplified bands from one MAW (ZGA2) and one PSC (ZGP5). The sizes of the PCR products matched the predicted sizes (315 to 387 bp) (Table 1). In total, we generated 435 clones with validated sequences including 234 from MAW and 201 from PSC. Alignment of all the sequences showed ten clusters (data not shown) representing ten genes. Figs. 2–4 show alignment s of intronic, exonic and amino acid sequences of ten gene representatives isolated from MAW ZGA2 and PSC ZGP5, respectively. The terminology for each subfamily follows previous studies [5], [8], [9], . Each pair specific to *EgAgB*1, 2 and 5 generated only one sequence cluster, respectively, indicating only one gene in the three subfamilies, comprising subfamily 1 (*EgAgB*1/1; accession numbers HM237302 (PSC) and GU166202 (MAW)), subfamily 2 (*EgAgB*2/1; accession numbers GU166200 (PSC) and GU166201 (MAW)) and subfamily 5 (*EgAgB*5/1; accession numbers GU166215 (PSC) and GU166216) (MAW)). In contrast, primers specific to subfamily 3 amplified four genes in the subfamily (*EgAgB*3/1–4, accession numbers GU166204-GU166214) whilst primers specific for *EgAgB* 4 generated three genes in the subfamily (*EgAgB*4/1–3, accession numbers GU166196-GU166199). Almost all the sequences in each gene cluster were identical to the *EgAgB* sequences deposited in GenBank (Figs. 2–4), which were obtained from isolates of *E. granulosus* from different geographical areas. Table 2 shows a comparison of each of the EgAgB DNA (intron and second exon) sequences and amino acid (aa) sequences encoded by the second exon, which are likely to be the mature and secreted proteins comprising 65–71 residues in length (Table S2). The degree of identity between the EgAgB protein family varies from 26.3% to 97.1%; the DNA sequences vary from 19% to 91% (Table 2). The lowest aa similarity occurred between EgAgB4/2 and EgAgB5/1. Although EgAgB3/1 has the highest identity (97.1%) with EgAgB3/2 at the aa level, the difference in their intronic sequences showed that they are different genes with a DNA identity of 57.1%.

**Table 2:** Identity matrix of *E. granulosus* antigen B family genes at the protein level encoded by the second exon and DNA level (italic).

Gene	B11	B21	B31	B32	B33	B34	B41	B42	B43	B51
B11		0.314	0.434	0.441	0.455	0.492	0.267	0.277	0.281	0.439
B21	*0.284*		0.315	0.319	0.347	0.338	0.633	0.666	0.69	0.3
B31	*0.392*	*0.286*		**0.971**	0.826	0.811	0.283	0.293	0.297	0.449
B32	*0.351*	*0.267*	*0.571*		0.852	0.838	0.287	0.297	0.301	0.455
B33	*0.213*	***0.19***	*0.242*	*0.24*		0.779	0.328	0.337	0.342	0.426
B34	*0.372*	*0.296*	*0.825*	*0.525*	*0.218*		0.319	0.328	0.333	0.507
B41	*0.355*	*0.48*	*0.348*	*0.287*	*0.224*	*0.324*		0.805	0.887	0.281
B42	*0.29*	*0.377*	*0.262*	*0.23*	*0.282*	*0.245*	*0.768*		0.859	**0.263**
B43	*0.355*	*0.463*	*0.354*	*0.28*	*0.222*	*0.327*	***0.912***	*0.735*		0.267
B51	*0.275*	*0.309*	*0.29*	*0.287*	*0.369*	*0.273*	*0.277*	*0.25*	*0.268*	

Note: protein sequence of *EgAgB* family genes: B11, EgAgB1/1; B21, EgAgB2/1; B31, EgAgB3/1; B32, EgAgB3/2; B33, EgAgB3/3; B34, EgAgB3/4; B42, EgAgB4/2; B43, EgAgB4/3; B51, EgAgB5/1; Avg, average; SD, standard deviation. The highest and lowest identity levels are bolded. DNA sequences include intronic sequences.

### *EgAgB* genes can be distinguished by variation in their intron and second exon sequences

The major differences between the *EgAgB* genes appear in their introns (Fig. 2) and the second exons (Fig. 3) which encode different protein sequences (Fig. 4). The intronic sequences can be used for distinguishing all subfamilies and four genes in the *EgAgB*3 subfamily as they have different sizes and variable sequence (Fig. 2). Based on alignment analysis with sequences from GenBank, *EgAgB1* has two clusters of intronic and exon sequences shown in Fig. S1. They are likely to be encoded by different alleles. However, in our study, only one unique sequence (*EgAgB1/1*) was amplified from individual worms and it has 89 bp of intronic sequence. The second exonic sequence comprises 198 bp (Fig. 3) encoding 65 aa (Table S2 and Fig. 4).

Cluster analysis of 99 cloned fragments of *EgAgB2* with intronic and the second exonic sequences isolated from individual PSC and MAW (30 sequences aligned in Fig. S2) showed that the subfamily *EgAgB2* comprises only one gene cluster, indicating there is only one gene in the *EgAgB2* subfamily. The intron is 68 bp in length and the second exon is composed of 213 bp encoding 70 aa (Table S2).

We designed a pair of primers to amplify the *EgAgB3* gene subfamily by PCR from genomic DNA. Based on the size of inserts in clones and subsequent sequence analysis, we isolated four clusters of fragments representing four genes in the subfamily. *EgAgB3/1*, *EgAgB3/2*, *EgAgb3/3* and *EgAgB3/4* had introns of 137 bp, 140 bp, 152 bp and 140 bp respectively (Fig. 2 and Table S2). Although EgAgB3/2 had the same sized intron (140 bp) as EgAgB3/4, there were 26 substitutions between the two sequences. The second exonic sequences of the *AgB3* subfamily also exhibited four types of sequences matching the intronic differences (Figs. 2–4). The amplified second exons of *EgAgB3/1* and *EgAgB3/3* encode 54 aa, but they are distinguishable from each other by differences in their intronic sequences of 137 and 152 bp, respectively (Fig. 2 and Table S2). In addition, there are eight aa substitutions in EgAgB3/1 compared with EgAgB3/3 (Fig. 4). The amplified regions of *EgAgB3/2* and *EgAgB3/4* encode 55 aa and 53 aa, respectively. The major difference in protein sequence encoded by the *EgAgB3* subfamily occurs in the region immediately linked to the signal peptide, which is a region rich in aspartic acid (D). EgAgB3/1 has 5Ds, EgAgB3/2 has 6Ds, EgAgB3/3 has 3Ds and EgAgB3/4 has 4Ds. Highly conserved sequences were found in the remainder of the second exonic sequences (Figs. 3, 4).

We designed four primers based on the 3′ terminal sequences of the *EgAgB4* subfamily including one for the 3′ UTR sequence. Combined with forward primer, EgAgBF2, the four pairs of primers allowed us to amplify three clusters of sequences from individual MAW, indicating that there are three genes (*EgAgB4/1*–*3*) present in the subfamily. *EgAgB*4 is very similar to *EgAgB*2 both in intronic and exonic sequence (Figs. 2, 3). The two subfamilies have the same sized 68 bp intron but there are ten nucleotide substitution differences in their intronic sequences (Fig. 2). In addition, there are 14–17 bp differences in the second exon of *EgAgB4* compared with *EgAgB2* (Fig. 3), resulting in 17 aa changes at the protein level (Fig. 4). The second exon of *EgAg4/3* is composed of 216 bp encoding 71 aa, while the second exons of *EgAgB 4/1* and *EgAgB4/2* encode 70 aa and 69 aa, respectively (Fig. 4). The intronic sequences of *EgAgB4* are identical. A major difference among the subfamily of genes is in their 3′ terminal exonic sequences, encoding different aa sequences rich in glutamic acid (E) and D residues (Fig. 4).

*EgAgB5* is a unique gene consisting of an intron of 67 bp and its second exon encodes a peptide of 66 aa (Fig. 4). Its DNA sequence is considerably different from those of the other *EgAgB* subfamily members (Table 2 and Figs. 2, 3). Consequently, the protein sequence of EgAgB5/1 has the lowest identity to the other proteins (Table 2).

### Phylogenetic analysis showing different evolutionary distance between *EgAgB* family genes

**Figure 5:**
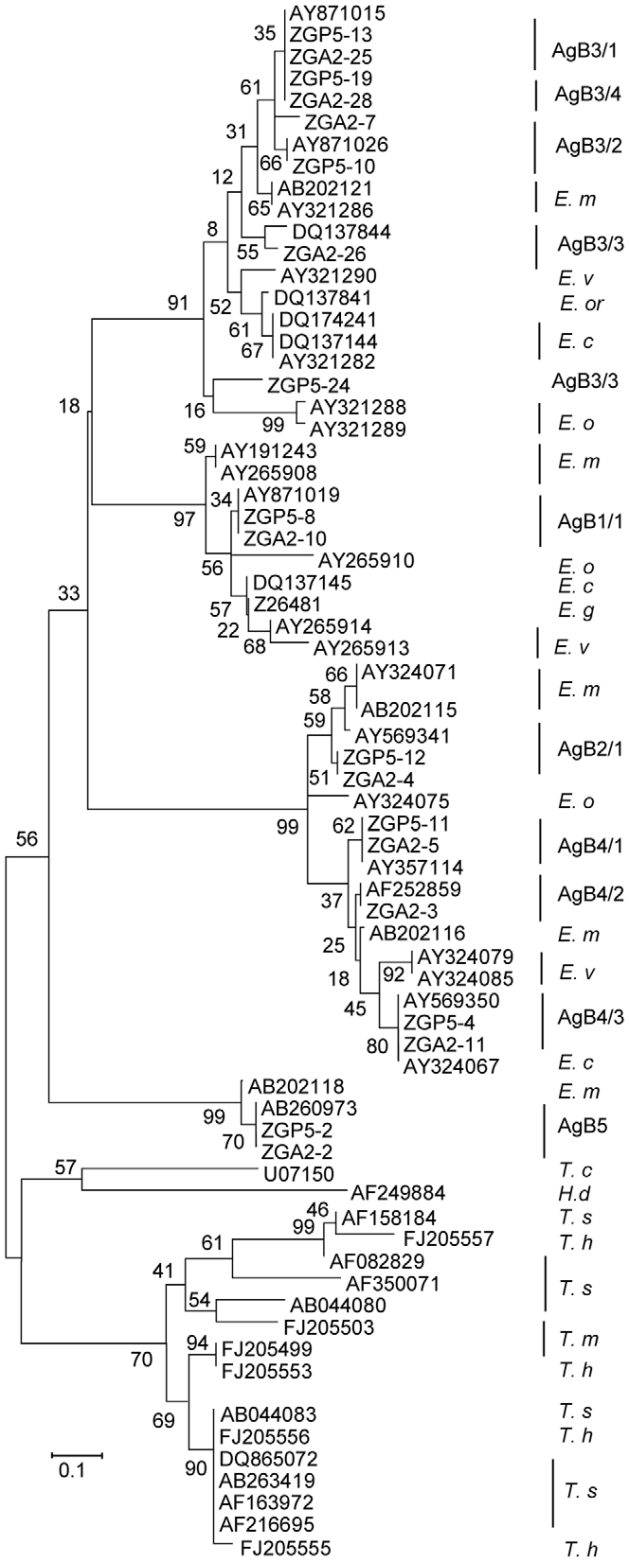
Phylogenetic analysis of ten *E. granulosus* antigen B family. Protein sequences encoded by the second exons of *EgAgB* genes isolated from a single PSC (ZGP) and MAW (ZGA), and homologues from other *Taenia* spp. and *Hymenolepis diminuta*. Sequences from GenBank with accession number were used for the tree analysis. Nucleotide sequence (with protein sequence) data reported in this paper are available in the GenBank, EMBL and DDBJ databases under the accession numbers: **GU166196**-**GU166216** with the same clone name shown in the figure. Bootstrap values (1000 replicates) are shown for each node.

We used MEGA methods for phylogenetic analysis of the inferred amino acid sequence of the *EgAgB* family of proteins to illustrate the evolutionary relationships within the family and, particularly, with those present in species from the confamilial genus *Taenia*. We confirmed the results with Bayesian analysis (Mr Bayes 3.1) [25] (data not shown) and the two methods showed a very similar evolutionary pattern. The minimum evolution tree (Fig. 5) has very low bootstrap values for deeper nodes, as anticipated because of dissimilarities between sequences from different subfamilies, and especially different species. The “Taeniidae antigens,” [26], commonly found in taeniid cestodes (and one example from *Hymenolepis diminuta*) form an outgroup in this mid-point rooted tree. All sequences from the genus *Echinococcus*, including sequences from *E. granulosus* (*EgAgB*), *E. multilocularis*, *E. vogeli*, *E. oligarthrus*, *E. ortleppi* and *E. canadensis* form a monophyletic clade (Fig. 5). This implies that these genes have radiated in the *Echinococcus* lineage after separation from the other taeniids.

For *Echinococcus*, the majority of the protein clusters include representative sequences from several species (Fig. 5), indicating the encoding genes were likely present in the most recent common ancestor of the genus suggesting the antigen B family has been important in its evolution.

### Genes differentially expressed in different stages of *E. granulosus*

It is important to note that we treated all RNA preparations for analysis with RNase-free DNase prior to reverse transcription. To determine whether the RNA samples contained DNA after treatment, we added a PCR control that comprised the cDNA synthesis reaction comprising all components but without the addition of reverse transcriptase (RT). Both normal RT and real-time PCR analysis showed there were no amplicons generated from these control samples (data not shown).

**Figure 6:**
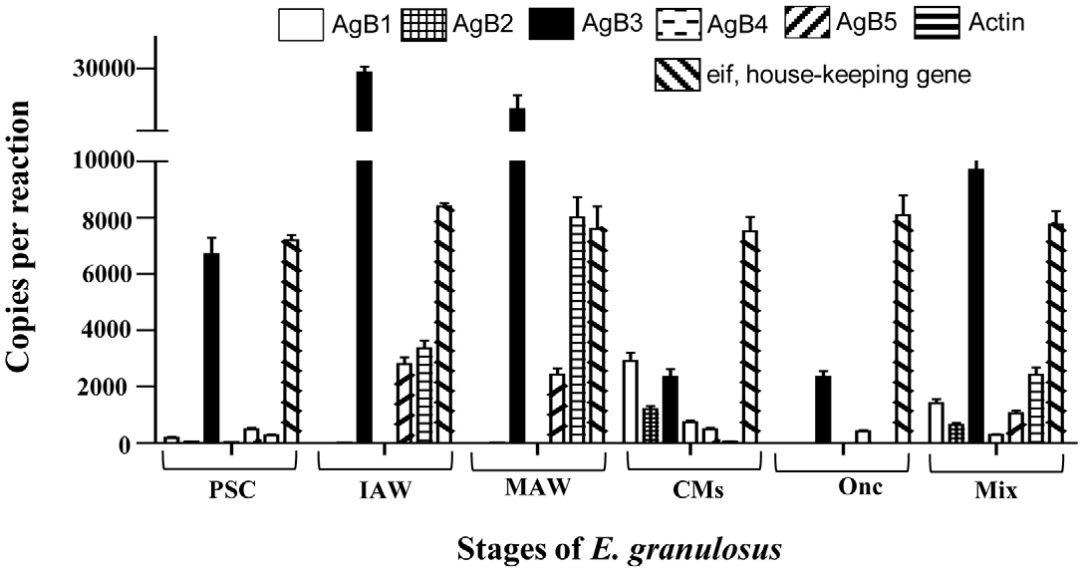
Expression levels of antigen B family in *E. granulosus* by real-time PCR. Genes representing five antigen B subfamilies (AgB1–5) were amplified using the subfamily specific primers with mRNA isolated from different stages of *E. granulosus*. PSC, protoscolex; CM, cyst membrane; IAW, immature adult worm; MAW, mature adult worm; ONC, oncosphere; Mix, a pooled sample of all stages. Actin, *E. granulosus actin* II. *E. granulosus* eukaryotic translation initiation factor (*Eg*-*eif*) was used as a house-keeping gene.

For normalizing the real time PCR data, we initially used *actin II* as a house-keeping gene to profile gene expression in the different stages of *E. granulosus*. However, as *actin II* was shown to be significantly up regulated in MAW and variable in the other stages, we used an eukaryotic translation initiation factor (*Eg*-*eif*) of *E. granulosus* as an alternative house-keeping gene, which was identified by microarray analysis and confirmed by real-time PCR and normal reverse transcription PCR analysis (data not shown). Figure 6 shows the results of the expression levels of 5 subfamilies of the *EgAgB* genes and *actin II* after normalization using *Eg-eif* in the 5 *E. granulosus* stages and a pooled mixture of the 5 stages as a PCR control with different combinations of primers. *EgAgB*1, *EgAgB2* and *EgAgB*5 were expressed at very low levels in all stages. *EgAgB3* was expressed in all stages of the parasite, with the highest in IAW and MAW. Except for *EgAgB3*, the *EgAgB* genes were almost undetectable in PSC and ONC. *EgAgB4* was expressed in CM, IAW and MAW, but at a low level. It is worth noting that *EgAgB3* was highly expressed in MAW (3–10 times higher than in the other stages), suggesting this gene subfamily may play a role in worm development in the gut of the definitive host. We used EgAgBF1 (Table 1) and EgAgB3R (Table S1) sequences as universal primers to amplify cDNA which showed (Table 3) that *EgAgB3/1* was the most highly expressed gene in all stages, followed by *EgAgB3/2*. *EgAgB3/3* and *EgAgB3/*4 were lowly expressed.

**Table 3:** Distributions of 4 Antigen B3 (AgB3) cDNAs in 30 clones randomly selected from each transformation of PCR products amplified from five stages of *E. granulosus*.

EgAgB	Stages				
	ONC	CW	PSC	IAW	MAW
AgB3/1	19	20	25	28	27
AgB3/2	11	10	4	2	3
AgB3/3	0	0	0	0	0
AgB3/4	0	0	1	0	0

Note: ONC, oncosphere; CW, hydatid cyst membrane; PSC, protoscolex; IAW, immature adult worm; MAW, mature adult worm.

## Discussion

All the genes in the *E. granulosus* antigen B (*EgAgB*) gene family have a similar gene structure with one intron flanked by two exons [27]. Furthermore, the first exonic sequence of *EgAgB* encodes a signal peptide. We did analysis of all *EgAgB* sequences deposited in the GenBank databases and showed that the sequences in this region are highly conserved (data not shown) with two clusters. This allowed us to design two forward primers, one for subfamily 1 and 3, and another for subfamily 2, 4 and 5 (Table 1) in the first exonic region of the gene family. The variable sequences occur at the 3′ terminal ends. Consequently, we designed eight downstream primers specific to the 3′ terminal sequences to cover all possible genes in the five recognised gene subfamilies. Primer EgAgB24UTR (Table 1) was designed based on the identical sequences of the 3′ terminal UTRs of subfamilies *EgAgB2* and *EgAgB4,* which allowed us to amplify the entire second exonic sequences in the subfamilies. With the designed primers, the PCR amplified fragments therefore contained both the intronic and the second exonic sequences of genes in the *EgAgB* family. Since eight pairs of primers were used to amplify genomic DNA from 11 MAW/PSC, instead of using random selection of clones for direct sequencing, we used a new strategy (described in detail in the Methods and Materials section) to select clones for sequencing. With this selection strategy, we chose 3–9 clones from each transformation for further sequencing. This strategy minimized the number of clones for sequencing and covered all possible *EgAgB* sequences. In total, we generated 435 clones with sequence information, which represents the largest reported number of *EgAgB* gene family sequences amplified from genomic DNA isolated from individual *E. granulosus* MAW and PSC. We isolated genomic DNA from individual PSC collected from a single hydatid cyst obtained from an infected kangaroo. The PSC clones allowed us to determine whether any apparent gene variation was caused by a different gene or by a mutation. As the PSC were collected from a single hydatid cyst, their genomic DNA should be identical [28], and, indeed, we showed the sequences for each gene were indistinguishable. Two conclusions resulted from this sequence analysis: 1). *E. granulosus* genomic DNA contains at least ten genes comprising the *EgAgB* family; and 2). each of the genes is highly conserved. We isolated all ten genes from each of six MAW. The MAW were collected from a dog experimentally infected with pooled PSC originating from a number of hydatid cysts obtained from three individual sheep. The worms could, therefore represent different genotypes, but the sequence analysis showed that each gene was identical, confirming, therefore, the conservation of each gene in the *EgAgB* gene family, which was further supported by alignment with sequences deposited in the GenBank databases (Fig. S3).

In addition, we showed that each of the ten *EgAgB* genes was identical in isolates collected from two distinct geographical areas, China and Australia. Macropods have only recently acquired *E. granulosus* as the parasite is believed to have been introduced into Australia by European immigrants about 200 years ago [29]. The conservation in sequence of the *EgAgB* genes isolated from a recently acquired new intermediate host, this case, a macropod, suggests that the *EgAgB* genes may play a fundamental role in parasite survival.

*EgAgB* has been considered to be a polymorphic gene [5], [8], due likely to host selection for adaption given that *E. granulsous* strains are generally specific for the intermediate hosts they infect [28]. Accordingly, different stains have been presumed to have different genomic isoforms or alleles for some of their *EgAgB* genes [10], [30]. An alignment with sequences from GenBank showed that *EgAgB1* has two or three major clusters of intronic and second exonic sequences (Fig. S1). As the sequences have the same intronic and exonic sequence lengths and several nucleotide substitutions, they are likely to be encoded by a polymorphic gene that is strain-related [31]. It is not clear whether the variation of the sequence is due to heterozygosity, which has been shown in the *Echinococcus* malate dehydrogenase (MDH) gene [32], or to the presence of host-specific alleles. We isolated one cluster of *EgAgB1* sequence from the MAW and larval PSC of *E. granulosus*. The parasite samples were collected from different hosts from two continents. One sequence (GU166203) was identical to one of the cluster sequences (AF143813 cluster, Fig. S1) that is related to a sheep strain sequence [31]. Another two clusters in the *EgAgB1* subfamily are related to those from *E. granulosus* cattle (FJ696924-FJ696928) and buffalo (FJ696936, FJ696923) strains [30], [31]. Further study is required to determine whether *EgAgB1* can be used as a universal probe for distinguishing the recognized genotypes of *E. granulosus* [33].

It is not surprising that *EgAgB* comprises a multigene family. Southern blotting analysis showed several bands present in hybridizations with genomic DNA from *E. granulosus* [8], [34] indicating the family has different genomic loci. With genomic DNA extracted from a single cyst, Chemale et al. [35] suggested there are three genes in the *EgAgB* gene family. Southern analysis, however, does not indicate precisely the number of genes in the family, which can only be determined by a sequencing approach.

We performed a phylogenetic analysis of inferred amino acid sequence of *EgAgB* family proteins to illustrate the evolutionary relationships within the family and particularly with those of the confamilial genus *Taenia* spp. (Fig. 5). The *Taenia* proteins have been termed “Taeniidae antigens,” as the encoding genes are found mostly in taeniid cestodes [26], with one sequence (AF249884) isolated from *Hymenolepis diminuta*, a member of the cyclophyllidean family Hymenolepididae. The proteins were classified into several major and distinct clusters. All sequences from the genus *Echinococcus*, including sequences from *E. granulosus* (EgAgB), *E. multilocularis*, *E. vogeli*, *E. oligarthrus*, *E. ortleppi* and *E. canadensis* form a monophyletic clade (Fig. 5), which is separated from those of the large tape worms, such as *Taenia* and *Hymenolepis.* This suggests that these genes have radiated in the *Echinococcus* lineage after its separation from the other taeniids. This radiation might be correlated with the unique biological features of the *Echinococcus* genus such as the extensive asexual reproductive capacity of the multi-compartmentalized metacestode stage, the use of different hosts and organs for cystic development, small MAW with few segments and low definitive host specificity; perhaps some or all of these traits are indicative of a role for the antigen B proteins.

Mumuti et al. [21] showed, using specific antibodies against each of 5 gene products in *E. multilocularis,* that the *EmAgB* genes were differentially expressed in the adult and larval cyst and PSC stages, with *EmAgB3* being predominantly expressed; however, the ONC stage, which is responsible for human infection, was not included in the analysis. To determine the expression of the *EgAgB* family of genes in *E. granulosus*, we used quantitative PCR to measure their expression levels in the PSC, CM, IAW, MAW and ONC. As the genes in each of the subfamilies have very similar sequences, it was challenging to design PCR primers to readily distinguish them individually. However, the differences in sequences between the subfamilies allowed us to design specific primers to amplify cDNA fragments to distinguish the genes at the subfamily level. We initially used *E. granulosus* actin II (accession no. L07773) as a house-keeping gene as used in other studies with *Echinococcus* [31], [36]–[39] but this gene proved to be highly variable between different stages of the parasite at the transcription level, being expressed 35 and 20 times higher in MAW than in the ONC and CM, respectively (Fig. 5). Our results showed that the *EgAgB* gene family members were expressed differentially, with the *EgAgB3* genes predominantly expressed in all life-cycle stages investigated, including the ONC. The expression profiles obtained were similar to these obtained by by Mamuti et al. [21], for *E. multilocularis*, who used specific antibodies against the EmAgB protein family. We were able to demonstrate that there are 4 genes in the *EgAgB3* subfamily. However, it is difficult to use normal real time PCR to distinguish their expression in *E. granulosus* due simply to the high similarity in their transcription levels. We expressed all the second exonic sequences of *EgAgB3* and subsequent analysis showed that they cross reacted strongly (data not shown), indicating neither normal real time PCR, nor Western blot analysis can be used for distinguishing each of the genes in the subfamily. Although not accurate, sequencing mRNAs from different stages of *E. granulosus* may be a way to predict the expression profiles of the *EgAgB3* genes based on the transcription frequency of the genes. We demonstrated that *EgAgB3/1* is the most predominant subfamily gene expressed in the intermediate host cyst and PSC stages, suggesting that EgAgB3/1 may be a suitable serodiagnostic target molecule.

It is almost 40 years since the EgAgB protein was identified in *E. granulosus* hydatid cyst fluid [40], but its precise biological function(s) still remains unknown. Here, we have shown that the *E. granulosus* antigen B family contains at least 10 genes. We believe these new findings are important for addressing the expression and regulation of the *EgAgB* genes, as they may provide new insights for determining the biological features and characteristics of the proteins encoded by this complex gene family, notably its potential role in the interaction between parasite and host as an immune modulator, benefiting parasite survival.

## Supporting Information

Table S1 (0.03 MB DOC)

Table S2 (0.04 MB DOC)

Figure S1 Comparison of variable regions of *E. granulosus* antigen B1. (A) Alignment comparison of the 3′ terminal second exonic sequences of antigen B1 subfamily with sequences deposited in the GenBank databases and a sequence ZGP5-8 (GenBank accession no HM237302) isolated from a PSC from a kangaroo hydatid cyst. Identical nucleotides to the first sequences (AF143813) are indicated with dots. Missing nucleotides are hyphenated. The second cluster of sequences is boxed. (B) Alignment comparison of intronic sequences of the antigen B1 subfamily with sequences deposited in the GenBank databases and a sequence ZGP5-8 isolated from a PSC from a kangaroo hydatid cyst. Identical nucleotides to ZGP5-8 are indicated with dots.
(0.19 MB TIF)

Figure S2 Alignment of EgAgB2/1 sequences isolated from *E. granulosus*. Thirty sequences were isolated from PSC (ZGP) and MAW (ZGA) of *E. granulosus* in the study showing that only one cluster existed in subfamily 2. The sequences in the first part are identical, which are not shown. Identical nucleotides to the first sequence (ZGA1B2-1) are indicated with dots.
(0.19 MB TIF)

Figure S3 Alignment comparison of protein sequences of *E. granulosus* antigen B. Ten *E. granulosus* antigen B protein sequences in the study are aligned with the sequences deposited in the GenBank databases. Identical amino acids to the first sequences are highlighted in black. Missing amino acids are hyphenated.
(0.70 MB TIF)
