# Supplementary material for: The Echinococcus granulosus Antigen B Gene Family Comprises at Least 10 Unique Genes in Five Subclasses Which Are Differentially Expressed
Source: PLoS Negl Trop Dis. 2010 Aug 10;4(8):e784. doi: 10.1371/journal.pntd.0000784 (PMC2919375; doi:10.1371/journal.pntd.0000784)
Supplement: Table S1 — (0.03 MB DOC) [file pntd.0000784.s001.doc]

Supplementary table 1 Primers used for real-time PCR

| Nmae | Sequences 5’→3’ | Size (bp) |
| --- | --- | --- |
| EgAgB1F | GATGATGGCCTCACCTCGAC |  |
| EgAgB1R | CCCTCTGAAGTGGGACCTGAG | 177 |
| EgAgB2F | GCCAAAAGCACACATGGG |  |
| EgAgB2R | TCTTAACATACTTCTTCAGCACCTCAC | 170 |
| EgAgB3F | GAAGTGACAAAGACGAAGAAGGGT |  |
| EgAgB3R | GTGCCTTCTTCCTCACCATCT | 142 |
| EgAgB4F | ACCCGAGAGATGCAAGTGC |  |
| EgAgB4R | CCTTGACATATTTCTTCAACACTTCGT | 137 |
| EgAgB5F | GACATCGATTCGAAAGCGAAG |  |
| EgAgB5R | AATCTCTTAAAGCCGATCGAGCT | 163 |
| EgActin II-F | GCCACTAGCTCCTCTCTGGA |  |
| EgActin II-R | GATGTCGAGATCGCACTTCA | 169 |

EgActin II, *E. granulosus* actin II, accession no. **L07773**.
