# Supplementary material for: The Echinococcus granulosus Antigen B Gene Family Comprises at Least 10 Unique Genes in Five Subclasses Which Are Differentially Expressed
Source: PLoS Negl Trop Dis. 2010 Aug 10;4(8):e784. doi: 10.1371/journal.pntd.0000784 (PMC2919375; doi:10.1371/journal.pntd.0000784)
Supplement: Table S2 — (0.04 MB DOC) [file pntd.0000784.s002.doc]

Supplementary table 2 Size of intron and the second exon of *E. granulosus* antigen B (*EgAgB*)

| Gene | Intron | Second exon (native) | |  | Second exon (amplified) | |
| --- | --- | --- | --- | --- | --- | --- |
|  | |  | |
|  | (bp) | DNA(bp) | Protein(aa) |  | DNA(bp) | Protein(aa) |
|  |  |  |  |  |  |  |
| *EgAgB1/1* | 89 | 198 | 65 |  | 186 | 62 |
| *EgAgB2/1* | 68 | 213 | 70 |  | 210 | 70 |
| *EgAgB3/1* | 137 | 210 | 68 |  | 163 | 54 |
| *EgAgB3/2* | 140 | 210 | 69 |  | 166 | 55 |
| *EgAgB3/3* | 152 | 201 | 68 |  | 163 | 54 |
| *EgAgB3/4* | 140 | 204 | 67 |  | 159 | 53 |
| *EgAgB4/1* | 68 | 213 | 70 |  | 210 | 70 |
| *EgAgB4/2* | 68 | 216 | 71 |  | 216 | 71 |
| *EgAgB4/3* | 68 | 210 | 69 |  | 210 | 69 |
| *EgAgB5/1* | 67 | 201 | 66 |  | 201 | 66 |

Note: bp, base pair; aa: amino acid.
