# Supplementary figures and images for: The Echinococcus granulosus Antigen B Gene Family Comprises at Least 10 Unique Genes in Five Subclasses Which Are Differentially Expressed
Source: PLoS Negl Trop Dis. 2010 Aug 10;4(8):e784. doi: 10.1371/journal.pntd.0000784 (PMC2919375; doi:10.1371/journal.pntd.0000784)

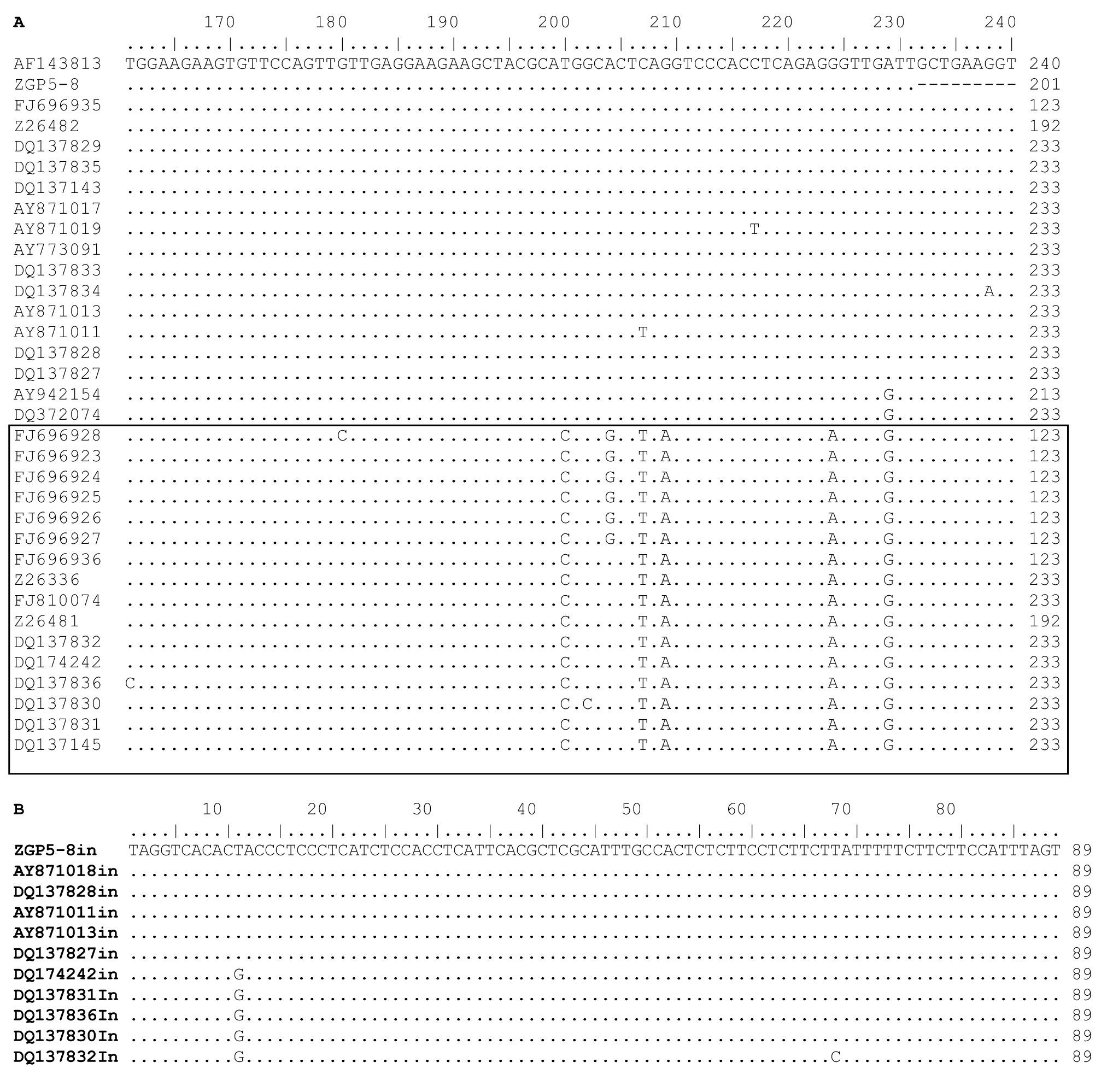

Supplement: Figure S1 — Comparison of variable regions of E. granulosus antigen B1. (A) Alignment comparison of the 3′ terminal second exonic sequences of antigen B1 subfamily with sequences deposited in the GenBank databases and a sequence ZGP5-8 (GenBank accession no HM237302) isolated from a PSC from a kangaroo hydatid cyst. Identical nucleotides to the first sequences (AF143813) are indicated with dots. Missing nucleotides are hyphenated. The second cluster of sequences is boxed. (B) Alignment comparison of intronic sequences of the antigen B1 subfamily with sequences deposited in the GenBank databases and a sequence ZGP5-8 isolated from a PSC from a kangaroo hydatid cyst. Identical nucleotides to ZGP5-8 are indicated with dots. (0.19 MB TIF) [file pntd.0000784.s003.tif]

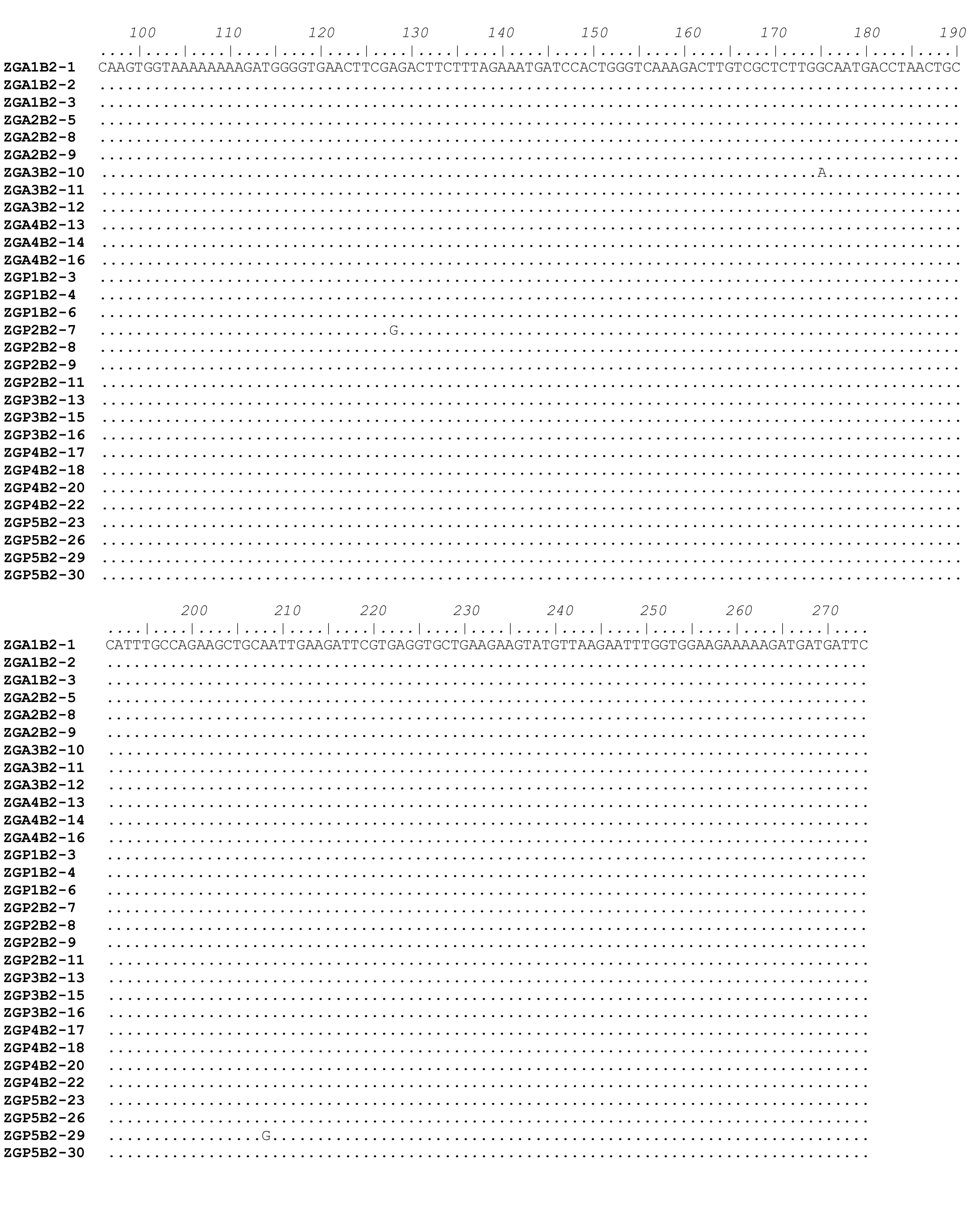

Supplement: Figure S2 — Alignment of EgAgB2/1 sequences isolated from E. granulosus. Thirty sequences were isolated from PSC (ZGP) and MAW (ZGA) of E. granulosus in the study showing that only one cluster existed in subfamily 2. The sequences in the first part are identical, which are not shown. Identical nucleotides to the first sequence (ZGA1B2-1) are indicated with dots. (0.19 MB TIF) [file pntd.0000784.s004.tif]

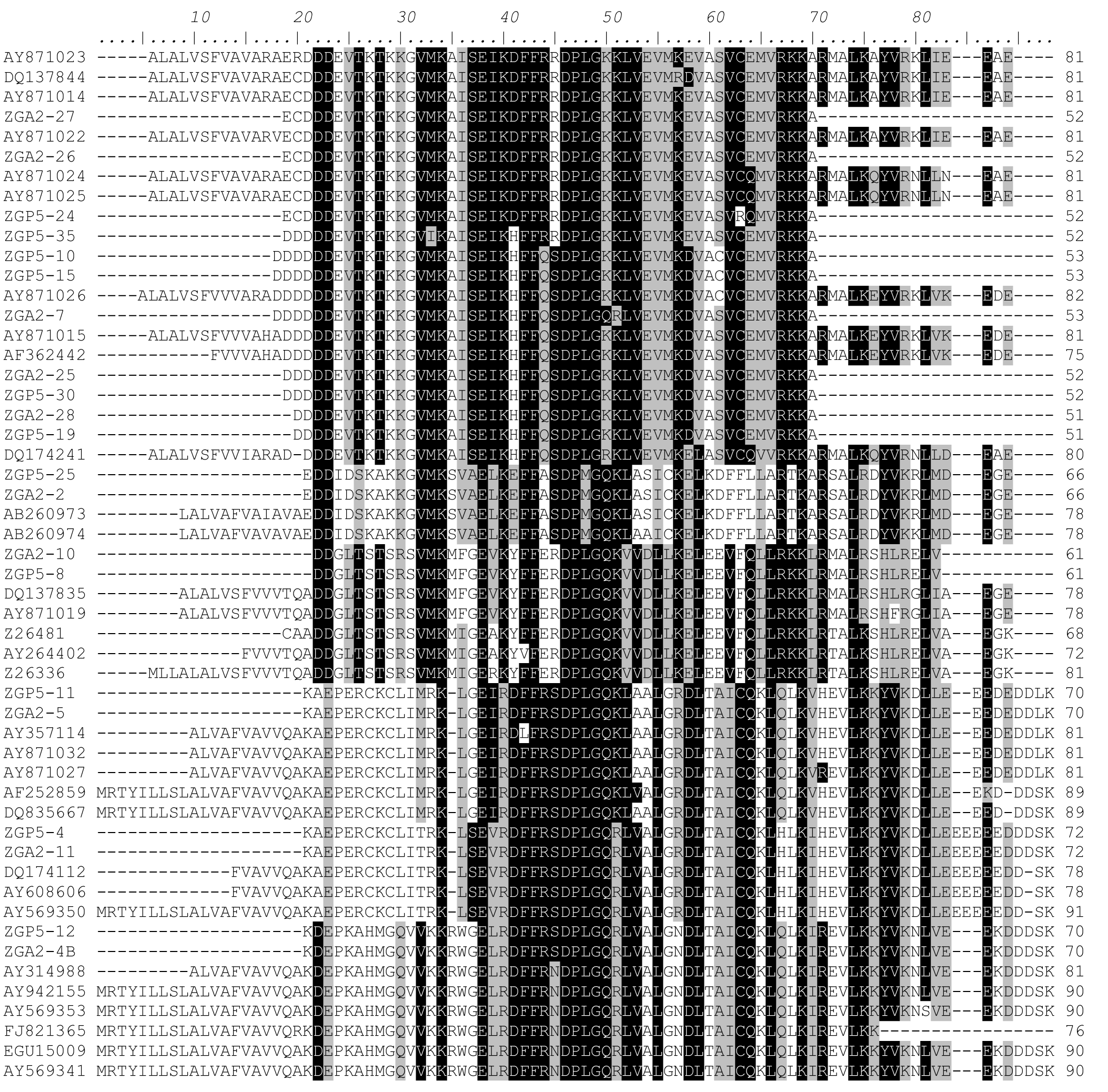

Supplement: Figure S3 — Alignment comparison of protein sequences of E. granulosus antigen B. Ten E. granulosus antigen B protein sequences in the study are aligned with the sequences deposited in the GenBank databases. Identical amino acids to the first sequences are highlighted in black. Missing amino acids are hyphenated. (0.70 MB TIF) [file pntd.0000784.s005.tif]
